# Gender differences in factors associated with prehypertension and hypertension in Nepal: A nationwide survey

**DOI:** 10.1371/journal.pone.0203278

**Published:** 2018-09-13

**Authors:** Kingsley Emwinyore Agho, Uchechukwu L. Osuagwu, Osita K. Ezeh, Pramesh Raj Ghimire, Stanley Chitekwe, Felix Akpojene Ogbo

**Affiliations:** 1 School of Science and Health, Western Sydney University, Campbelltown Campus, Penrith, New South Wales, Australia; 2 School of Medicine | Diabetes Obesity and Metabolism Translational Research Unit (DOMTRU), Macarthur Clinical School, Campbelltown, New South Wales, Australia; 3 United Nations Children Funds (UNICEF), United Nations House, Pulchowk, Lalitpur Nepal; 4 Translational Health Research Institute, School of Medicine, Western Sydney University, Campbelltown Campus, Penrith, New South Wales, Australia; 5 Prescot Specialist Medical Centre, Welfare Quarters, Makurdi, Benue State, Nigeria; The University of Warwick, UNITED KINGDOM

## Abstract

**Background:**

Nepal has one of the highest prevalence of hypertension in South Asia. However, no national studies have examined the gender differences in the determinants of prehypertension and hypertension in the country to inform targeted interventions. This study aimed to investigate gender differences in factors associated with prehypertension and hypertension in Nepal using the 2016 Nepal Demographic and Health Survey (NDHS).

**Methods:**

Sociodemographic, behavioural, anthropometric and health status data and information on hypertension were obtained from 14,857 (males: 6,245 and females: 8,612) individuals aged 15 years or above from the biomarker sample of the 2016 NDHS. Factors associated with prehypertension and hypertension by gender were investigated using generalized linear latent and mixed models (GLLAM) with the mlogit link and binomial family that adjusted for clustering and sampling weights.

**Results:**

The overall prevalence of prehypertension and hypertension was 26.9% [95% confidence interval (CI): 25.7, 28.1] and 17.2% (95% CI 16.1, 18.3), respectively. Prehypertension was present in 30.4% (95%CI: 28.7, 32.2) of males and 24.3% (95% CI: 23.1, 25.6) of females, while hypertension was present in 20.4%, (95% CI 18.9, 22.0) of males and 14.8% (95% CI: 13.7, 16.0) of females. Key modifiable factors that were strongly associated with prehypertension and hypertension in both genders included overweight and obesity, caffeine intake, tobacco use, no schooling, previously informed of hypertension in a health facility, and alcohol consumption (for males). Other significant factors associated with prehypertension and hypertension included increasing age (> 30 years), ecological zone (Hill), Developmental zone (Western) and being married.

**Conclusion:**

Our results suggest that prehypertension and hypertension were higher in males compared to females. Interventions to improve awareness, screening, treatment and control of prehypertension and hypertension in Nepal are warranted and should target key modifiable factors, as well as people aged 30 years and above.

## Introduction

Hypertension is a global public health issue that accounted for an estimated 141 deaths and 2,869 disability-adjusted life years (DALYs) per 100,000 population in 2016 [1–4]. Similarly, an estimated 874 million adults had hypertension in 2015, with an increase expected within the next decade [1]. Globally, in 2015, hypertension was the leading risk factor for deaths and health loss, largely from ischemic heart disease, haemorrhagic and ischaemic stroke [1]. Based on the increasing burden of hypertension and other non-communicable diseases (e.g., diabetes mellitus and cancers), the 66th World Health Assembly endorsed the World Health Organisation Global Action Plan for the Prevention and Control of Non-Communicable Diseases (NCDs) 2013–2020 [5]. The main aim of this strategic plan is to galvanise a multi-sectoral collaboration at the national, regional and global levels to reduce the avoidable burden of morbidity, mortality and disability due to NCDs. Achieving reductions in hypertension and its complications would require high-quality research to promote and support preventive programs. Similarly, the assessment of determinants of hypertension by gender is also warranted to guide targeted and high-impact initiatives [5].

Previous studies in human and animal populations have shown that differences in hypertension exist in males and females due to biological and behavioural factors [6–9], where males had higher levels of hypertension compared to females [7, 9]. The biological factors of hypertension are poorly understood, but sex hormones and genetic differences have been shown to predispose to hypertension [10, 11]. Behavioural factors included high body mass index [12], high salt intake [13] and low physical activity [12, 14]. The mechanism as to how these behavioural factors differentially predispose to hypertension in both males and females is rather complex [15], with studies suggesting the involvement of inflammatory cells and the activation of the renin-angiotensin–aldosterone system [16–18]. Environmental determinants such as temperature, season, latitude and altitude have also been flagged as contributing to the onset of hypertension, but whether these factors differ by gender remain inconclusive [19, 20].

Recent estimates indicate that low- and middle-income countries continue to have increased burden of hypertension compared to high-income countries [21–23]. The 2016 Nepal Demographic and Health Survey (NDHS) revealed that the prevalence of hypertension varied among males (22.0%) and females (15.0%) [24]. Similarly, a nationwide survey on NCDs conducted in 2013 found that 26.0% of Nepalese population aged 15–69 years had hypertension [25]. Previous hospital-based studies and reports from regional areas of Nepal have suggested that overweight and obesity, older age, male gender, alcohol consumption, physical inactivity, high blood glucose level, increased total cholesterol and cigarettes smoking were associated with hypertension [22, 26–29]. However, evidence from these studies is limited, wherein they may not be generalised to the broader Nepalese population to inform national level strategic actions. Additionally, to the authors’ knowledge, no nationwide studies have previously examined gender variations in factors associated with prehypertension in Nepal.

The assessment of determinants of prehypertension and hypertension by gender is essential for identifying health gaps, planning and implementation of effective public health control and prevention strategies in high-risk groups and communities in Nepal. We aimed to investigate gender differences in factors associated with prehypertension and hypertension in Nepal, using the 2016 NDHS, nationally representative household data.

## Methods

The health and socio-demographic data for the current study were extracted from the 2016 NDHS conducted by the Nepalese Ministry of Health in conjunction with the United States Agency for International Development via Inner City Fund (ICF) International. The detailed statistical procedure used in gathering data and procedure for blood pressure (BP) measurements have been described elsewhere [24]. Briefly, information on BP was available from weighted 14,857 eligible survey participants aged 15 years and older, consisting of 8,612 women and 6,245 men. Before the BP measurements, informed consent was sought from the adult respondents aged 18 years and above, while consent for those aged between 15 and 17 years was provided by their parents or another adult in the household at the time of the survey. Respondents were also informed about the reasons and procedure for the BP measurement including strict confidentiality of the BP outcome. Automated digital UA-767F/FAC (A&D Medical) BP monitors were used, and each of the participants was measured in line with internationally recommended categories [24]. Participation in BP measurements in Nepal household survey was high in both men and women, yielding a response rate of 95% and 97%, respectively [24].

### Study outcome

The study outcome consists of three variables (normal, prehypertension and hypertension) after a respondent BP was measured against the standardised level of cut-off points for BP range [24]. A respondent aged 15 years and above was considered to be hypertensive if the systolic BP (SBP) was ≥ 140 mmHg and/or diastolic BP (DBP) was ≥ 90 mmHg. A respondent on anti-hypertensive prescription medication at the time of the survey was also classified as hypertensive. A respondent was categorised as prehypertensive if SBP ranged between 120 mmHg and 139 mmHg, and/or DBP ranged between 80 mmHg and ≤ 89 mmHg, and/or on anti-hypertensive medication during the survey. A respondent was classified as having a normal BP if SBP was <120 and/or DBP < 80 mmHg [24].

In this study, we used a multinomial approach for the outcome variables such that a respondent was considered *normal* (coded as ‘0’) if BP measurement lies in the specified range, *prehypertensive* (coded as ‘1’) if BP measurement lies in the specified range, or *hypertensive* (coded as ‘2’) if BP measurement lies in the specified range.

### Exposure variables

The possible confounding variables considered in the current study were influenced by a previous study on prehypertension and hypertension [30] and data availability. We categorised these variables into five groups: community-level factors, socio-demographic level factors, behavioural level factors, anthropometric level factors, and health service level factor. The community-level factors assessed were type of residence, ecological zone, developmental region and state categorised into seven provinces.

The socio-demographic level factors investigated included household wealth index, mother’s education, father’s education level, mother’s age and marital status. The household wealth status was estimated based on household facilities and assets available to participants at the time of the survey, using a principal component analysis [31]. Household wealth status was classified into five groups: poorest, poorer, middle, richer and richest. Behavioural factors examined were tobacco use, alcohol use and caffeine intake. The tobacco use (cigarette smoking) was examined in the analysis because a prospective cohort study of cigarette smoking and risk of hypertension in women indicated that women who had at least 15 cigarettes per day had increased risk of developing hypertension [32]. Also, a study from England showed a significant relationship between cigarette smoking and hypertension in both males and females [33]. Alcohol use was assessed in the analysis because a study by Maheswaran et al. [34] revealed that alcohol intakes significantly elevated systolic and diastolic BP in both men and women, particularly when consumed 1 to 3 days before BP measurement.

Body mass index (BMI) measurement was based on weight in kilograms (kg) and height in square meters (m^2^) of respondents interviewed during the survey. BMI is primarily utilised in estimating the level of obesity or underweight in a population. Based on previous findings of a significant association between BMI and blood pressure elevation in adults [18, 35], we incorporated BMI, and it was classified into three groups: BMI is < 18.5 (underweight), BMI is 18.5 to < 24.9 (normal), or BMI is > 25 to 34.9 (overweight and obesity). We also included a health service factor, that is, information on previous knowledge of hypertension in the study analysis.

### Statistical analysis

**Fig 1:**
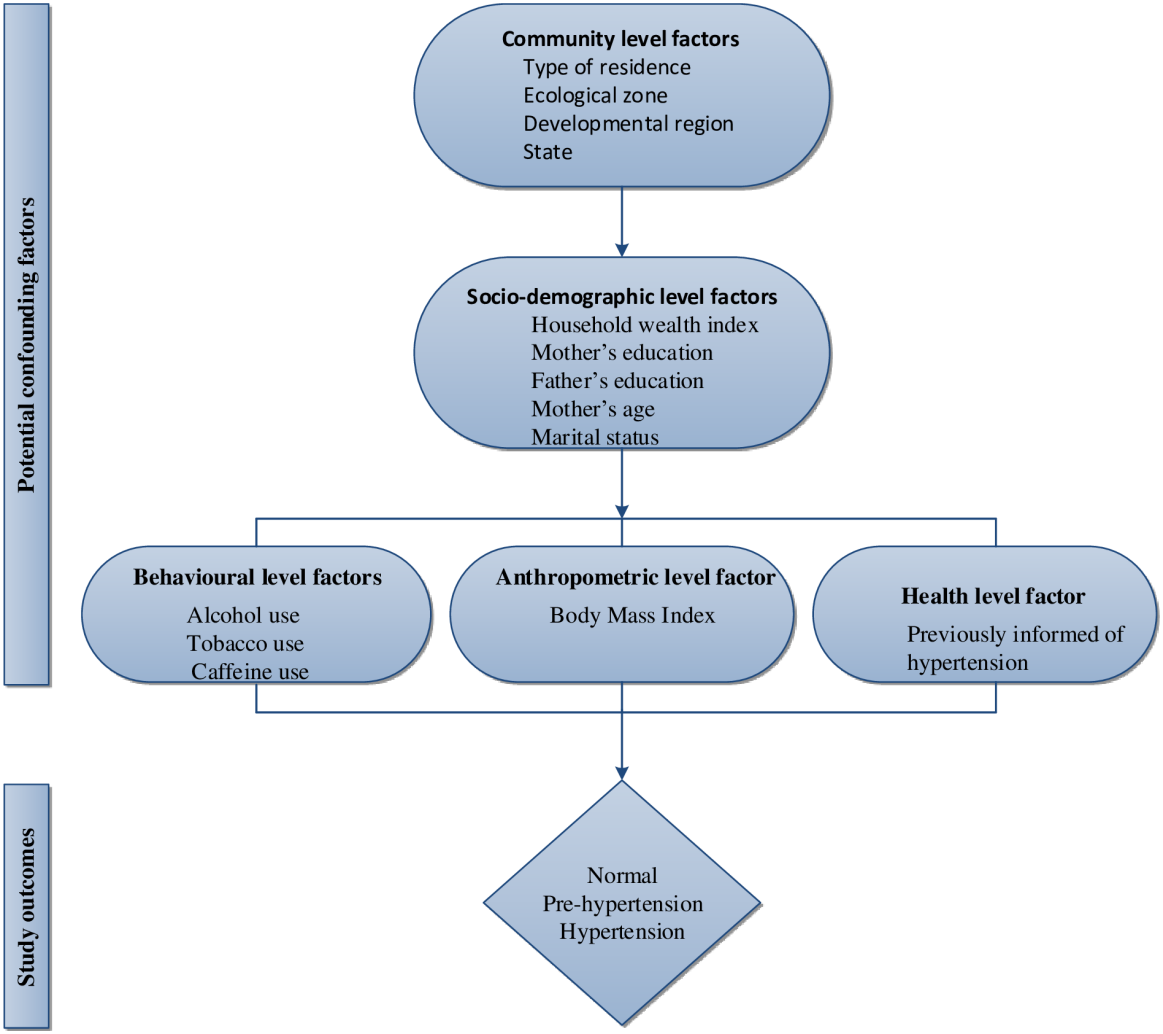
Contextual framework for analysing factors associated with prehypertension and hypertension among males and females aged 15 years and over in Nepal.

‘Svy’ commands were employed to allow for adjustments for the cluster-sampling design and weight. First, frequency tabulations were conducted to describe the data, and this was followed by the contingency table analyses to examine the factors associated with prehypertension and hypertension by gender. Taylor series linearization method was used in the analyses when estimating confidence intervals (CIs) around the point estimates. Second, univariate models that utilised generalized linear latent and mixed models (GLLAM) with the mlogit link and binomial family that adjusted for clustering and sampling weights were calculated to determine the unadjusted odds ratios of factors associated with prehypertension and hypertension by males and females. Third, a multivariable model that used generalized linear latent and mixed models (GLLAM) with the mlogit link and binomial family that adjusted for clustering and sampling weights was performed in a five-stage model to investigate the adjusted odds ratio of factors associated with prehypertension and hypertension by gender [Fig 1].

In the first model, community-level factors were entered into the model. A manually executed elimination method was conducted to determine factors associated with prehypertension and hypertension (by males and females) at a 0.05 significance level. In the second model, the significant factors in the first stage were added to socio-demographic factors, and this was followed by elimination procedure. A similar approach was used for behavioural, anthropometric and health service factors in the third, fourth and fifth stages, respectively.

In the final model, we tested and reported any co-linearity. The odds ratios with 95% confidence intervals were calculated to assess the adjusted risk of independent variables. All analyses were carried out using STATA/MP version 14 (Stata Corp, College Station, TX, USA).

### Ethics

The DHS project obtained ethical approval from the Nepal Health Research Council-Kathmandu before the surveys were conducted. The data used in this study were anonymous and are publicly available to apply for online. Approval was sought from Measure DHS/ICF International and permission was granted for this use.

## Results

### Characteristics of the study population

The present analysis included 14,857 (males = 6,245 and females = 8,612) weighted total of individuals aged 15 and above from the NDHS, 2016. Nearly two-thirds of the respondents (9,113 participants) lived in the urban areas, and more than a third of the respondents (5,745 individuals) lived in the rural areas. Males (56%) had more secondary or higher level of education compared to females (39%). Females were more overweight or obese compared to males (22% and 17%, respectively) in the study [Table 1].

**Table 1:** Characteristics of study variables by males and females in Nepal, 2016.

Study variables	Female (N = 8,612) n(%)	Male (N = 6,245) n(%)
*Community level factors*		
**Type of residence**		
Urban	5257(61)	3856(62)
Rural	3355(39)	2390(38)
**Ecological zone**		
Mountain	545(6)	405(6)
Hill	3811(44)	2731(44)
Terai	4256(50)	3109(50)
**Developmental region**		
Eastern	2029(24)	1446(23)
Central	3038(35)	2379(38)
Western	2009(23)	1430(23)
Mid-Western	921(11)	606(10)
Far Western	614(7)	384(6)
**State**		
province 1	1515(18)	1105(18)
province 2	1726(20)	1305(21)
province 3	1825(21)	1414(22)
province 4	900(10)	632(10)
province 5	1433(17)	991(16)
province 6	450(5)	310(5)
province 7	760(9)	485(8)
*Household level factors*		
**Household wealth index**		
Poorest	1575(18)	1091(17)
Poorer	1710(20)	1172(19)
Middle	1772(21)	1206(19)
Richer	1847(21)	1413(23)
Richest	1708(20)	1363(22)
**Education level**		
Secondary and higher	3356(39)	3489(56)
Primary	1183(14)	1269(20)
No education	4068(47)	1482(24)
*Individual factors*		
**Current age**		
15–29	929(11)	587(9)
30–44	2779(32)	1886(30)
45–69	4262(50)	3239(52)
70+	641(7)	533(9)
**Marital status**		
Never married	1375(16)	1410(23)
Ever married	7237(84)	4835(77)
*Behaviour factor*		
**Alcohol use**		
No	8558(99)	6083(97)
Yes	53(1)	162(3)
**Tobacco use**		
No	8322(97)	5052(81)
Yes	290(3)	1193(19)
**Caffeine use**		
No	8106(94)	5634(90)
Yes	506(6)	611(40)
*Anthropometric factor*		
**BMI(kg/m**^**2**^**)**		
Underweight	1228(14)	823(13)
Normal	5472(64)	4335(70)
Overweight and obesity	1876(22)	1046(17)
*Health factor*		
**Previously informed of hypertension**		
No	7700(89)	5471(88)
Yes	912(11)	774(12)

Notes: n (%), a weighted sample of men and women whose blood pressure was measured (and proportion) across each variable; BMI, body mass index; Kg, kilogramme; m^2^, squared meters

**Fig 2:**
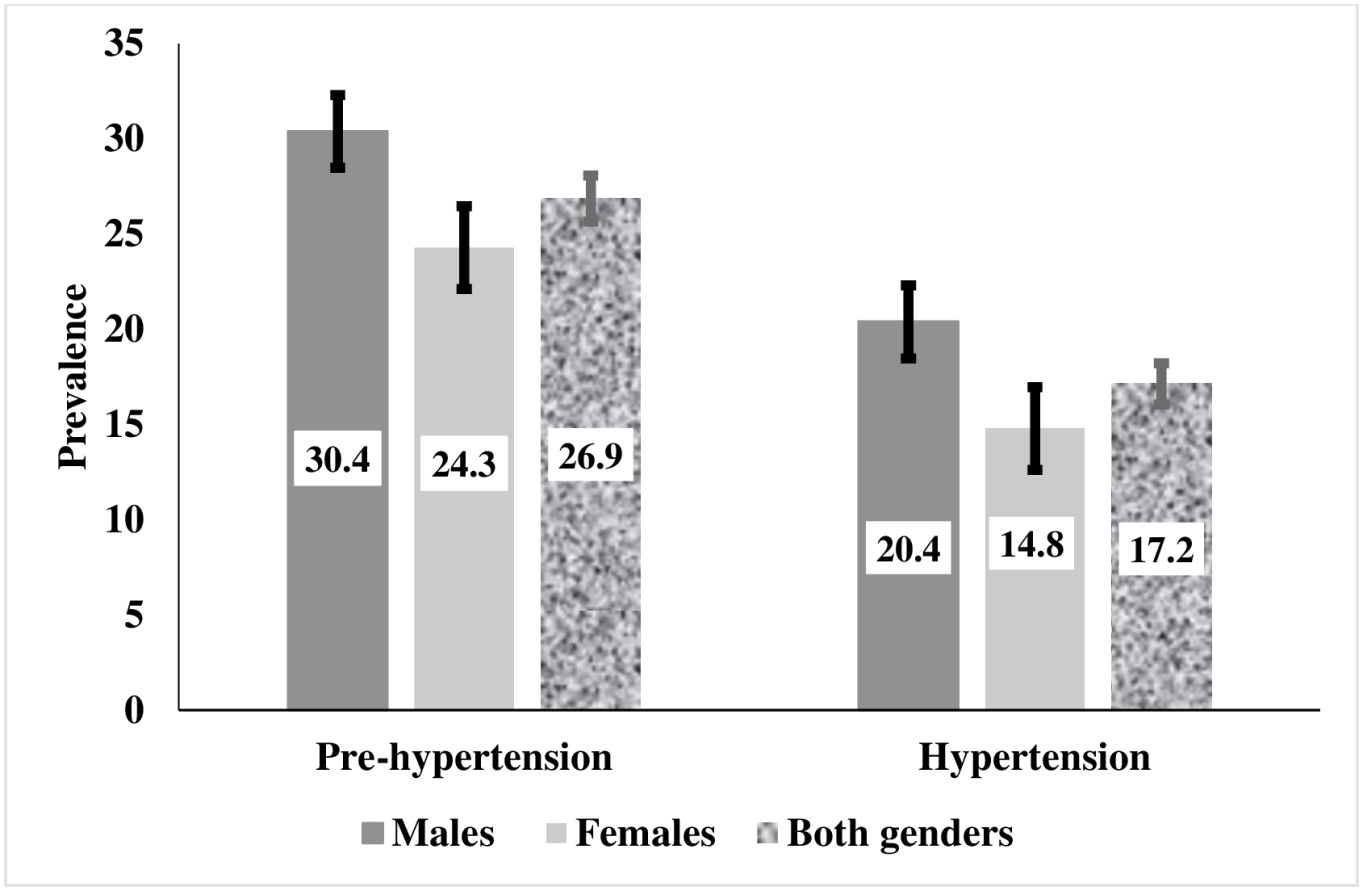
Prevalence of prehypertension and hypertension by gender in Nepal (NDHS, 2016). (A) Males; (B) Females; (C) Both genders.

As illustrated in Fig 2, males were significantly more likely to be prehypertensive (30.4% vs. 24.3%, P < 0.001) and hypertensive (20.4% vs. 14.8%, P < 0.001) compared to females. In both genders, the overall prevalence of prehypertensive and hypertensive differ statistically by males but did not differ statistically by females.

### Determinants of prehypertension and hypertension among females

Females aged 15 years and above who lived in the Hill and Terai Ecological zone and those who lived in the Western developmental region were more likely to experience prehypertension and hypertension [Table 2]. Females with no schooling or completed primary and those aged 45 years and above had higher odds of experiencing prehypertension and hypertension compared to females who completed secondary or higher level of education and younger age females (15–44 years), respectively.

**Table 2:** Determinants of prehypertension and hypertension among females in Nepal, 2016.

Study variables	Prehypertension		Hypertension	
	Unadjusted	Adjusted	Unadjusted	Adjusted
	OR(95% CI)	AOR(95% CI)	OR(95% CI)	AOR(95% CI)
*Community level factors*				
**Type of residence**				
Urban	1.00		1.00	
Rural	1.01(0.86, 1.19)		0.98(0.82, 1.17)	
**Ecological zone**				
Mountain	1.00	1.00	1.00	1.00
Hill	1.39(1.01, 1.91)	1.71(1.20, 2.42)	1.33(0.95, 1.88)	1.67(1.13, 2.47)
Terai	1.08(0.79, 1.49)	1.47(1.02, 2.12)	0.94(0.66, 1.32)	1.34(0.89, 2.03)
**Developmental region**				
Eastern	1.00	1.00	1.00	1.00
Central	1.25(1.01, 1.54)	1.22(0.96, 1.55)	1.02(0.81, 1.29)	1.02(0.78, 1.35)
Western	1.55(1.24, 1.93)	1.59(1.22, 2.06)	1.68(1.33, 2.13)	1.94(1.46, 2.59)
Mid-Western	0.99(0.76, 1.30)	0.95(0.70, 1.30)	0.67(0.49, 0.91)	0.76(0.53, 1.10)
Far Western	0.95(0.70, 1.29)	0.98(0.69, 1.38)	0.67(0.46, 0.96)	0.86(0.57, 1.29)
**State**				
province 1	1.00		1.00	
province 2	0.90(0.70, 1.17)		0.68(0.51, 0.91)	
province 3	1.28(0.98, 1.67)		1.21(0.91, 1.60)	
province 4	1.69(1.28, 2.24)		1.90(1.41, 2.56)	
province 5	1.26(0.97, 1.64)		1.30(0.98, 1.72)	
province 6	0.88(0.63, 1.22)		0.54(0.35, 0.81)	
province 7	0.82(0.61, 1.11)		0.56(0.39, 0.79)	
*Household level factors*				
**Household wealth index**				
Poorest	1.00	1.00	1.00	1.00
Poorer	0.96(0.80, 1.14)	0.99(0.81, 1.20)	1.09(0.88, 1.34)	1.04(0.82, 1.32)
Middle	0.90(0.75, 1.09)	0.94(0.76, 1.17)	0.89(0.71, 1.11)	0.86(0.66, 1.12)
Richer	0.85(0.70, 1.02)	0.86(0.69, 1.08)	0.87(0.69, 1.09)	0.79(0.60, 1.04)
Richest	0.93(0.75, 1.14)	0.84(0.65, 1.08)	1.27(1.01, 1.61)	0.86(0.63, 1.16)
**Education level**				
Secondary and higher	1.00	1.00	1.00	1.00
Primary	1.54(1.29, 1.83)	1.24(1.03, 1.49)	2.12(1.70, 2.64)	1.49(1.16, 1.91)
No education	3.05(2.68, 3.47)	2.38(2.05, 2.76)	5.22(4.44, 6.13)	3.50(2.88, 4.26)
*Individual factors*				
**Current age**				
15–29	1.00	1.00	1.00	1.00
30–44	1.55(1.27, 1.89)	1.35(1.10, 1.66)	1.58(1.22, 2.05)	1.24(0.93, 1.64)
45–69	1.63(1.35, 1.97)	1.33(1.09, 1.63)	2.24(1.75, 2.86)	1.46(1.11, 1.91)
70+	2.01(1.55, 2.61)	1.50(1.14, 1.97)	3.33(2.44, 4.55)	1.86(1.31, 2.64)
**Marital status**				
Never married	1.00	1.00	1.00	1.00
Ever married	2.62(2.23, 3.08)	1.29(1.07, 1.55)	7.86(5.83, 10.60)	2.30(1.66, 3.20)
*Behaviour factor*				
**Alcohol use**				
No	1.00		1.00	
Yes	1.50(0.74, 3.03)		3.19(1.64, 6.18)	
**Tobacco use**				
No	1.00	1.00	1.00	1.00
Yes	2.94(2.21, 3.92)	2.05(1.52, 2.78)	3.37(2.46, 4.61)	1.92(1.34, 2.73)
**Caffeine use**				
No	1.00	1.00	1.00	1.00
Yes	1.93(1.54, 2.40)	1.60(1.26, 2.03)	2.45(1.92, 3.12)	1.64(1.24, 2.18)
*Anthropometric factor*				
**BMI(kg/m**^**2**^**)**				
Underweight	1.00	1.00	1.00	1.00
Normal	1.30(1.11, 1.54)	1.46(1.22, 1.73)	1.22(0.99, 1.51)	1.41(1.12, 1.78)
Overweight and obesity	3.17(2.61, 3.84)	3.37(2.72, 4.17)	4.93(3.92, 6.21)	4.53(3.46, 5.94)
*Health factor*				
**Previously informed of hypertension**				
No	1.00	1.00	1.00	1.00
Yes	3.66(2.98, 4.49)	2.79(2.25, 3.46)	16.67(13.78, 20.16)	11.19(9.10, 13.77)

Note: 95% confidence intervals [CI] that include 1.00 indicate a non-significant resul; BMI, body mass index; Kg, kilogram; m^2^, squared meters

Females who smoked tobacco and consumed any caffeine-related drinks were more likely to experience prehypertension and hypertension compared to those who did not smoke tobacco, or consumed any caffeine-related drinks, respectively. Females who were previously informed of hypertension and those who were overweight and obese were more likely to experience prehypertension and hypertension compared to those who were not previously informed of hypertension and underweight, respectively.

**Fig 3:**
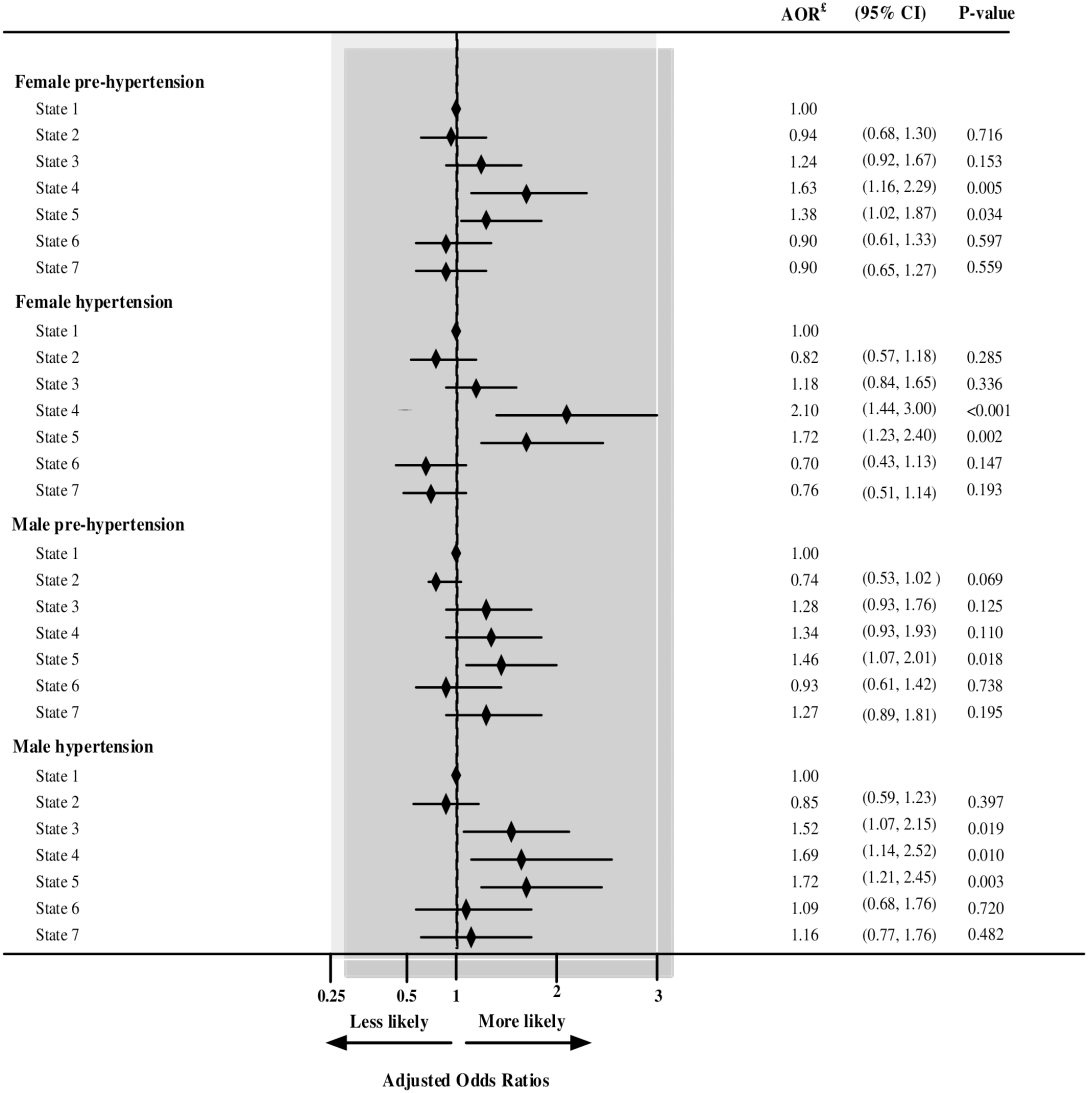
Prehypertension and hypertension among males and females aged 15 years and over in seven states in Nepal. ^**£**^ Independent variables adjusted for: type of residence, ecological zone, developmental region, household wealth index, education level, current age, marital status, alcohol use, tobacco use, caffeine use, BMI (kg/m^2^), and previously informed of hypertension.

To test for collinearity in the final model for females, we replaced Development zone with States, and our result showed that States 4 and 5 were associated with prehypertension and hypertension (Fig 3).

### Determinants of prehypertension and hypertension among males

Males who lived in rural areas were less likely to experience hypertension compared to males who lived in the urban areas. Males aged 15 years and above who lived in the Hill Ecological zone and in the Western Developmental region were more likely to experience prehypertension and hypertension compared to their counterparts who lived in the Mountain and Terai Ecological zones, and Eastern Developmental region. In comparison to younger males (15–29 years), males aged 30 years and above were more likely to experience hypertension [Table 3].

**Table 3:** Determinants of prehypertension and hypertension among males in Nepal, 2016.

Study variables	Prehypertension		Hypertension	
	Unadjusted	Adjusted	Unadjusted	Adjusted
	OR(95% CI)	AOR(95% CI)	OR(95% CI)	AOR(95% CI)
*Community level factors*				
**Type of residence**				
Urban	1.00	1.00	1.00	1.00
Rural	0.95(0.79, 1.14)	0.93(0.77, 1.12)	0.47(0.42, 0.53)	0.77(0.62, 0.95)
**Ecological zone**				
Mountain	1.00	1.00	1.00	1.00
Hill	1.59(1.13, 2.23)	1.70(1.18, 2.45)	2.05(1.41, 2.98)	2.16(1.43, 3.27)
Terai	1.03(0.74, 1.45)	1.14(0.79, 1.64)	1.14(0.78, 1.66)	1.21(0.80, 1.84)
**Developmental region**				
Eastern	1.00	1.00	1.00	1.00
Central	1.07(0.84, 1.37)	1.04(0.81, 1.34)	1.31(1.01, 1.71)	1,17(0.89, 1.54)
Western	1.64(1.28, 2.12)	1.56(1.19, 2.04)	2.03(1.55, 2.66)	1.90(1.41, 2.56)
Mid-Western	1.14(0.84, 1.55)	0.85(0.68, 1.31)	1.16(0.83, 1.63)	0.89(0.61, 1.28)
Far Western	1.20(0.84, 1.70)	1.24(0.86, 1.79)	1.02(0.68, 1.52)	1.11(0.72, 1.70)
**State**				
province 1	1.00		1.00	
province 2	0.90(0.70, 1.17)		0.68(0.51, 0.91)	
province 3	1.28(0.98, 1.67)		1.21(0.91, 1.60)	
province 4	1.69(1.28, 2.24)		1.90(1.41, 2.56)	
province 5	1.26(0.97, 1.64)		1.30(0.98, 1.72)	
province 6	0.88(0.63, 1.22)		0.54(0.35, 0.81)	
province 7	0.82(0.61, 1.11)		0.56(0.39, 0.79)	
*Household level factors*				
**Household wealth index**				
Poorest	1.00		1.00	
Poorer	0.97(0.78, 1.19)		1.18(0.93, 1.50)	
Middle	0.93(0.74, 1.16)		0.97(0.75, 1.25)	
Richer	1.00(0.81, 1.25)		1.04(0.82, 1.33)	
Richest	1.25(0.99, 1.57)		1.69(1.32, 2.17)	
**Education level**				
Secondary and higher	1.00	1.00	1.00	1.00
Primary	1.21(1.03, 1.42)	0.97(0.82, 1.16)	1.61(1.35, 1.93)	1.23(1.01, 1.50)
No education	1.25(1.07, 1.46)	1.01(0.85, 1.20)	1.79(1.51, 2.12)	1.35(1.10, 1.65)
*Individual factors*				
**Current age**				
15–29	1.00	1.00	1.00	1.00
30–44	1.43(1.14, 1.79)	1.20(0.95, 1.52)	2.06(1.54, 2.77)	1.53(1.11, 2.10)
45–69	1.68(1.35, 2.08)	1.40(1.12, 1.75)	2.69(2.03, 3.57)	1.91(1.41, 2.60)
70+	1.76(1.31, 2.37)	1.32(0.97, 1.80)	3.69(2.61, 5.23)	2.03(1.38, 2.99)
**Marital status**				
Never married	1.00	1.00	1.00	1.00
Ever married	2.68(2.46, 3.34)	2.11(1.78, 2.50)	9.69(7.52, 12.48)	5.12(3.90, 6.73)
*Behaviour factor*				
**Alcohol use**				
No	1.00	1.00	1.00	1.00
Yes	1.65(1.11, 2.46)	1.45(0.96, 2.20)	2.04(1.35, 3.09)	1.65(1.05, 2.60)
**Tobacco use**				
No	1.00	1.00	1.00	1.00
Yes	1.53(1.30, 1.79)	1.31(1.10, 1.55)	1.97(1.67, 2.34)	1.50(1.24, 1.81)
**Caffeine use**				
No	1.00	1.00	1.00	1.00
Yes	1.37(1.11, 1.69)	1.16(0.93, 1.44)	1.95(1.56, 2.42)	1.45(1.14, 1.85)
*Anthropometric factor*				
**BMI(kg/m**^**2**^**)**				
Underweight	1.00	1.00	1.00	1.00
Normal	2.08(1.70, 2.53)	1.90(1.55, 2.33)	1.80(1.42, 2.27)	1.52(1.18, 1.95)
Overweight and obesity	6.22(4.83, 8.00)	4.76(3.65, 6.21)	9.66(7.32, 12.75)	5.75(4.23, 7.82)
*Health factor*				
**Previously informed of hypertension**				
No	1.00	1.00	1.00	1.00
Yes	3.40(2.71, 4.28)	2.55(2.01, 3.24)	10.52(8.46, 13.09)	6.80(5.39, 8.58)

Note: 95% confidence intervals [CI] that include 1.00 indicate a non-significant result; BMI, body mass index; Kg, kilogram; m^2^, squared meters.

Ever married males and those who were overweight and obese were more likely to be prehypertensive and hypertensive compared to never married males and those with underweight, respectively. Males who reported alcohol and tobacco used, consumed any caffeine-related drinks and were previously informed of hypertension were more likely to be pre-hypertensive and hypertensive compared to their counterparts who use no alcohol and tobacco, did not consume any caffeine-related drinks and were not previously informed of hypertension, respectively.

In the final model for men, when Development zone was removed from the final model and replaced with States, we found that State 4 was significantly associated with prehypertension, while States 4 and 5 were associated with hypertension (Fig 3).

## Discussion

Our study found that prehypertension and hypertension were more prevalent among males (30.4% and 20.4%) than females (24.3% and 14.8%), respectively, consistent with other reports from Nepal [22, 24, 27]. Common key modifiable factors that were strongly associated with prehypertension and hypertension in both genders included overweight and obesity, caffeine intake, no schooling, previously informed of hypertension in a health facility, and alcohol consumption (for males). Other significant factors associated with prehypertension and hypertension included increasing age (> 30 years), ecological zone (Hill), Developmental zone (Western) and being married. Smoking has been documented as a risk factor for elevated blood pressure in both males and females [32, 33, 36, 37], and our study showed that tobacco smoking was associated with prehypertension and hypertension in both genders.

The finding that the consumption of any caffeinated drinks was associated with prehypertension and hypertension in Nepalese population underpins the ongoing debate on the link between caffeine intake and hypertension. Evidence from observational studies [38–42], randomised controlled trials [43, 44] and a systematic review [45] have shown mixed results on the relationship between caffeine intake and the onset of hypertension. For example, studies from Australia [38], Spain [39] and France [42] indicated that caffeine intake was related to hypertension in both males and females. In contrast, a report from Japan [43] and meta-analysis [46] showed that caffeine intake through coffee lowered blood pressure. A recent study from Spain found that caffeine intake was not associated with increased risk of hypertension [47]. The variations in the epidemiologic evidence on the impact of caffeine intake on hypertension may be due to the methodology used for measuring caffeine intake, including the quantity and duration of intake.

The prevalence of hypertension increases with age in all populations [21, 27, 29, 37, 48–50], with males more likely to be affected [29, 48, 50]. Similarly, our study indicated that the odds of developing prehypertension and hypertension were higher in respondents aged 30 years and above, with males representing a higher proportion. This may be attributed to the higher health-risk behaviour such as tobacco use (19% vs 3%) and use of alcohol (3% vs 1%) in males compared to females in Nepalese population [24], or genetic predisposition [10]. In Nepal, it would be beneficial for adults (and males) to be screened regularly for prehypertension and hypertension by health professionals, and cases of prehypertension and hypertension should be appropriately investigated and those confirmed should be educated on preventive and management strategies to avoid hypertension-related mortality and disability.

In the current study, the use of alcohol in males was associated with hypertension, while tobacco smoking in both males and females was associated with prehypertension and hypertension. Evidence has indicated that cigarette smoking increases the risk of hypertension [32, 33], and in a recent study conducted in Nepal [29], current and past tobacco smoking were associated with hypertension. Previous studies [51, 52] have not only demonstarted the association between tobacco smoking and alcohol intake and elevated blood pressure but also their impact on other NCDs such as cancers, stroke and pulmonary diseases. The Nepal Health Sector Strategy 2016–2021 will be essential in reducing hypertension and its complications as it seeks to reduce the prevalence of hypertension by 4% by the year 2020.

The adverse effect of overweight and obesity in the human population are well-documented [53–55]. Our study found that overweight and obesity were associated with prehypertension and hypertension in both males and females, consistent with previous studies from Nepal [21, 29]. The mechanism as to why high body mass index increases the risk for elevated blood pressure include increased resistance to insulin and retention of salt [56] and decreased physical activity [57]. Reports from Nepal have suggested that watching television for more than 2 hours per day [58, 59], high socioeconomic status and consuming fruit four times or less per week were strongly associated with obesity [58]. For many years, researchers and health promoters have proposed measures to reduce the burden of obesity, including taxation to limit intake of unhealthy foods, subsidising the consumption of healthy foods and restricting advertisement of unhealthy foods to children [60, 61]. The Nepalese government at all levels would do well to consider implementing those evidence-based policies and programmes to reduce overweight and obesity and thus, reduce hypertension and other NCDs.

The present study indicated that urbanization and residence in the Western region of Nepal were associated with prehypertension and hypertension in both genders. This was consistent with evidence from previous studies [29, 37, 48, 49], which found that place of residence was associated with hypertension. In a study from China, urbanization accounted for more than half (57.7%) of the variation between provinces in hypertension status [49]. We observed that individuals who lived in States 4 and 5 reported higher odds of prehypertension and hypertension compared to individuals who lived in State 1. States 4 and 5 represented individuals from the Hill and the Terai ecological regions [9] related to higher odds of prehypertension and hypertension compared to those who lived in the Mountainous region. Our findings were supported by the research conducted by Thapa and colleagues who concluded that individuals from Dalit and Janajati ethnic background were more likely to consume alcohol-related drinks compared to their upper caste counterparts [62]. Differences in ethnic groups regarding unhealthy behaviours like tobacco smoking, use of alcohol and physical inactivity [27, 29] may explain our findings of regional differences in the prevalence of hypertension. Additional epidemiological studies are needed to elucidate the influence of regional/state on the onset of prehypertension and hypertension in Nepal.

The study has a number of limitations. First, an assessment of other significant risk factors of hypertension such as family history of hypertension, history of diabetes, physical inactivity, salt intake, diet and HDL-cholesterol level would have provided a more robust assessment of determinants of prehypertension and hypertension. Second, the measurement of blood pressure was conducted in one day for participants, and some of the respondents with hypertension may not be clinically classified as prehypertensive or hypertensive. Third, the type, duration and quantity of alcohol consumed or tobacco use are important limitations to considered as a lack of the information may have over- or underestimated the point estimate. Fourth, caution should be exercised when interpreting the findings (particularly those for Central development region) given that the survey was conducted a year after the huge 7.8 magnitude earthquake that hit the country in 2015, leaving widespread impact on the Nepalese population [24]. Fifth, our study was based on cross-sectional data, and the establishment of a temporal relationship between the study factors and the outcomes cannot be established.

Despite these limitations, for the first time, this study provides nationally representative evidence on gender variations in the determinants of prehypertension and hypertension in Nepal. Also, selection bias is unlikely to affect the study findings given the high response rate in both males (95%) and females (97%). The use of trained and experienced staff, as well as a standardized protocol for the blood pressure measurement in populations (three separate measurements), improved the accuracy of the blood pressure readings, and the sex-specific data were also strengths of this study.

## Conclusion

The current study showed that prehypertension and hypertension were more common among males compared to females in Nepal. Significant modifiable determinants of prehypertension and hypertension included overweight and obesity, tobacco smoking, intake of caffeine-related drinks, and consumption of alcohol (for males). The local adoption of the WHO action plan on NCDs, including interventions to increase awareness, screening, treatment and control of elevated BP is warranted to improve health outcomes of the Nepalese population.
